# Exophytic Squamous Cell Carcinoma of the Scalp: A Case Report

**DOI:** 10.7759/cureus.36741

**Published:** 2023-03-27

**Authors:** Cole J Homer, Tony Richa

**Affiliations:** 1 Otolaryngology - Head and Neck Surgery, University of Nebraska Medical Center, Omaha, USA

**Keywords:** oncology, otolaryngology, scalp, exophytic, squamous cell carcinoma, case report

## Abstract

Squamous cell carcinoma (SCC) is the second-most common clinical presentation of non-melanoma skin cancer. Despite its prevalence, the rate of growth and development of SCC lesions is low. We present a case report of an exophytic SCC of the midline scalp. Over approximately 18 months, the exophytic portion of this lesion grew to a size of 8.5 x 7 cm due to the fact that the patient did not seek medical attention. The patient suffered from many predisposing factors including active smoking status, type II diabetes, and significant previous sun exposure. In addition to these predisposing factors, the patient did not have comprehensive health insurance to cover outpatient medical care. This case highlights the importance of early intervention in the management of head and neck skin cancers and the negative impact of delayed treatment.

## Introduction

Squamous cell carcinoma (SCC) is a neoplastic proliferation of the squamous epithelium. SCC is the second-most common category of non-melanoma skin cancer, with European studies reporting an incidence range of 9-96 per 100,000 in males and 5-68 per 100,00 in females [1-3]. Although SCC is prevalent, tumor growth and metastasis rates are relatively low: a US analysis revealed an average risk of nodal metastasis of 3.0% and a risk of disease-specific death of 2.1% [4]. The most common clinical presentations of SCC lesions are skin, head, neck, esophagus, and non-small cell lung cancers [5]. Additionally, the larynx is another of the most common sites for the presentation of SCC [6]. One England-based study reported that the median age at presentation of cutaneous SCC diagnosis was 80 years [7].

In addition to SCC, other lesions may also involve the scalp and demonstrate a similar clinical presentation. A recent Taiwanese study found that the most common types of local skin cancer were basal cell carcinoma (BCC) and SCC [8]. Following these skin lesions, scalp tumors' third most common presentation was due to metastasis from a distant primary tumor, with the lung being the most common source [8]. The optimal treatment method for a scalp lesion depends heavily upon the clinical circumstances and the specific type of lesion. Localized skin cancers, such as BCC or SCC, may be able to be treated with local excision alone. On the other hand, distant metastases or significant malignant skin cancers of the scalp may require surgical intervention at the site of the primary tumor and/or chemoradiation of the entire body.

The most significant risk factors that can lead to the development of cutaneous SCC are sun exposure, fair skin, increased age, and immunosuppression [9]. If identified early, SCC can be treated with surgery or radiation alone [10,11]. With more advanced diseases, however, resection with or without adjuvant chemoradiation may be required [10,11]. This article presents a case of extensive, neglected, exophytic SCC of the scalp.

## Case presentation

A 68-year-old male presented with an extensive, neglected, exophytic SCC of his midline scalp. Informed consent was obtained for the purposes of this case report. The patient was unemployed and underinsured with a risk factor history of type II diabetes, smoking, and significant sun exposure.

**Figure 1:**
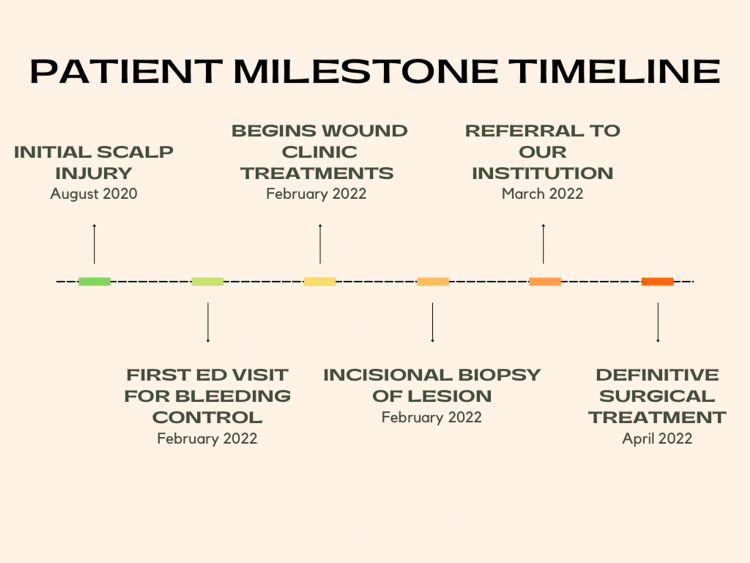
Patient milestone timeline

The patient described experiencing a traumatic injury to his scalp after accidentally bumping his head into a cabinet corner approximately 18 months prior to presentation, which then developed into a persistent non-healing ulcerative lesion. Over the next few months, the lesion developed a slowly growing exophytic component. Two weeks before presenting to our head and neck surgery clinic, the patient started to experience uncontrolled, persistent bleeding from the mass, prompting him to seek care from his local ED. The patient made four additional visits to the ED due to concerns about bleeding from the tumor, and he was referred to a local wound care clinic. While under the care of the wound clinic, the patient underwent an incisional biopsy of the lesion that histologically revealed atypical squamous proliferation. Figure 1 shows the patient milestone timeline.

**Figure 2:**
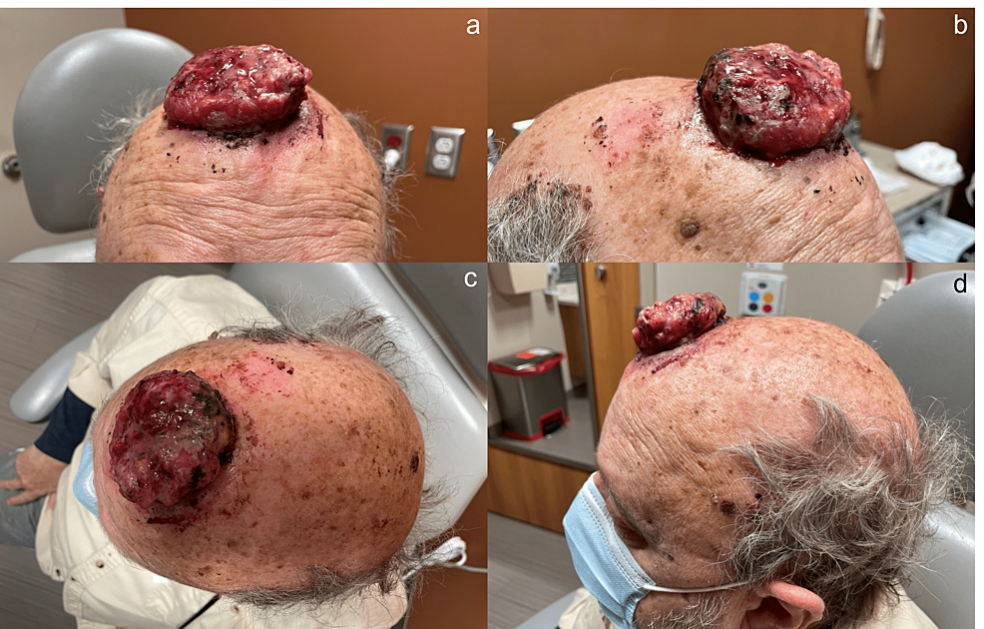
Pre-surgical presentation of lesion at time of initial referral to our institution

Following this biopsy, the patient reported a significant increase in the lesion size, and he was subsequently referred to our institution's head and neck surgery clinic (Figure 2).

**Figure 3:**
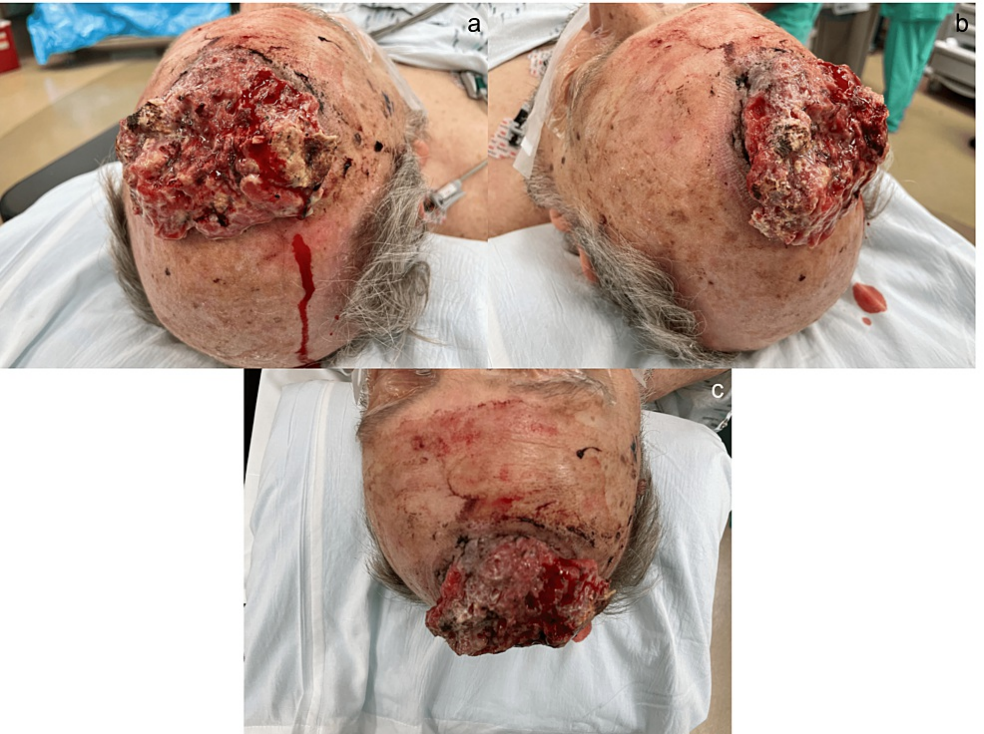
Presentation of lesion at time of surgical resection

Upon presentation to our head and neck surgery clinic, the patient’s physical examination demonstrated an exophytic, indurated, tender, and ulcerated mass of the midline anterior scalp with a diameter of approximately 5 cm and multiple bleeding areas (Figure 3).

The mass was mobile at the time of examination, and there were no palpable cervical lymph nodes. The patient’s outside incisional biopsy was reviewed at our institution and showed SCC. A CT scan of the head and neck with contrast was obtained, which revealed an “enhancing mass involving the scalp and extending into the subcutaneous fat, measuring up to 7.7 cm.” There was no evidence of regionally metastatic disease or infiltration of the calvarium. The patient’s case was presented at our institutional multidisciplinary head and neck tumor board, and the recommendation was made to proceed with surgical resection of the mass with local scalp reconstruction.

**Figure 4:**
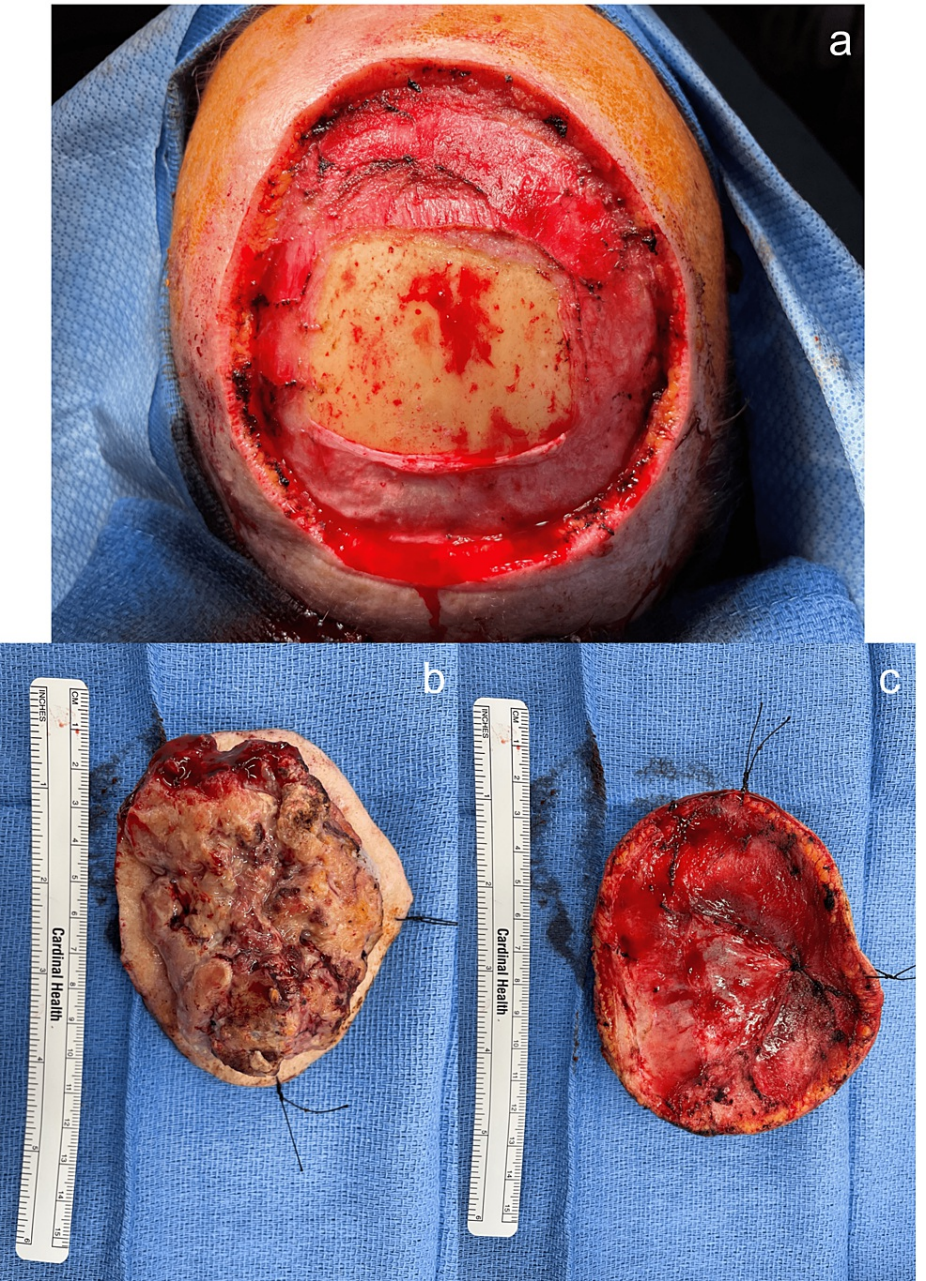
Intraoperative presentation following excision of the lesion demonstrating the (a) remaining tissue following excision, (b) excised lesion, and (c) internal side of excised lesion demonstrating periosteal involvement

The surgical intervention was performed 49 days after the patient’s initial presentation to our institution and 60 days after his presentation to the ED at which this lesion was first medically reported. The delay in the surgical intervention was due to difficulty with financial clearance for the procedure. During this delay, the patient continued to visit his local ED for bleeding control, and the lesion continued to increase in size. On the day of surgery, the final exophytic growth of the lesion measured approximately 8.5 x 7 cm. The patient underwent a wide local excision of his scalp SCC with a 1 cm circumferential macroscopically clear margin. The dissection proceeded with care taken to remain deep to the subgaleal fascia. However, during the resection, it was noted that the epicenter of the lesion extended to and involved the underlying periosteum. The involved periosteum was incised with a 1 cm macroscopically clear margin and was carefully elevated off the underlying calvarium. The resected periosteum was kept in continuity with the initial cutaneous resection specimen. The underlying calvarial bone was then carefully examined and was determined not to be involved with the disease process. The resection specimen was then sent for a frozen section for evaluation of circumferential and en-face margins. The frozen section confirmed all margins were clear of disease. The final cutaneous scalp defect measured 10 x 10 cm, and the underlying periosteal defect measured 5 x 5 cm (Figure 4).

**Figure 5:**
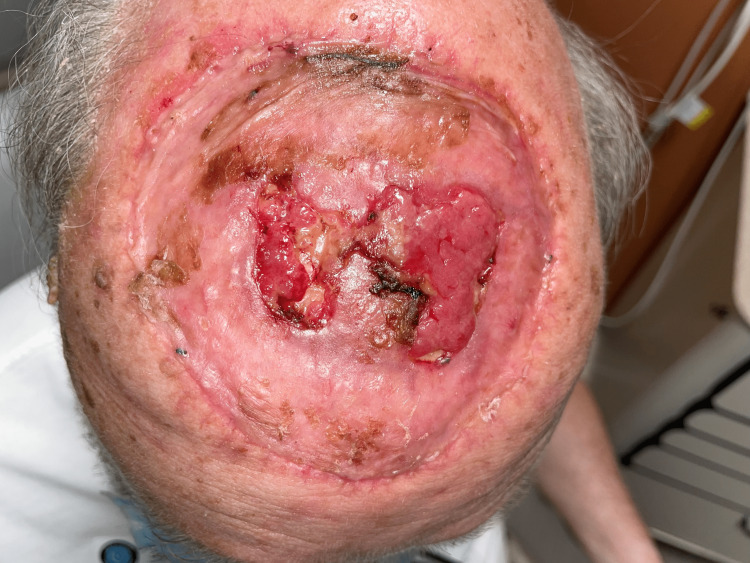
Patient skin graft at four-week post-operatively

The resection defect was then copiously irrigated with sterile saline. Reconstruction of the scalp defect and coverage of the exposed calvarial bone was planned with a split-thickness skin graft from the left anterior thigh. The superficial cortical bone of the exposed calvarium was drilled down to expose healthy bleeding vessels to prepare the area to receive the split-thickness skin graft. Using a dermatome with a 4-inch (10.2 cm) blade, a 0.17-inch thickness split-thickness graft was harvested from the left anterior thigh. The split-thickness skin graft was then transferred to cover the scalp defect and sutured in place using 4-0 chromic gut sutures. This provided excellent coverage of the defect with no exposed calvarium. A Xeroform bolster was placed to cover the split-thickness skin graft and held in place with 2-0 silk sutures. The patient was then admitted to the inpatient ward. He had an uneventful postoperative recovery and was discharged home the next day. The bolster was removed a week later in the clinic, and the clinical exam demonstrated a healthy viable skin graft with more than 90% take. Final pathology demonstrated SCC with no lymphovascular invasion (LVI) or perineural invasion (PNI) and clear surgical margins. The patient was again presented at our multidisciplinary tumor board to discuss adjuvant treatment. The decision was made to proceed with close surveillance in light of negative surgical margins and no adverse features of the tumor (no LVI or PNI). At the patient’s four-week follow-up visit, his skin graft had healed nicely with the development of healthy granulation tissue (Figure 5).

The patient continues close oncologic surveillance every three months with repeat neck ultrasounds.

## Discussion

There are several factors that, when combined, function to explain how this case of SCC of the scalp could develop as extensively as it did in this patient. First, the patient was 68 years old with an extensive history of UV sun exposure on the scalp; both are known risk factors for developing SCC [9]. The patient also reported that the lesion developed from a traumatic injury which resulted in a persistent non-healing ulcer. In addition to the risk factors listed above, the patient has a history of smoking and type II diabetes, both of which have also been shown to interrupt the healing process and increase the risk of SCC [12-14].

While many of these risk factors may contribute to the development of SCC, the extensive growth of this lesion is due in large part to the patient’s limited access to medical services and preventive care. In the United States, the majority of health insurance takes the form of private health insurance, with employer-sponsored plans serving as a common route for acquiring private health insurance. In addition to private health insurance, the United States also has federally funded health insurance programs in place known as Medicare (patients aged 65 years and over or with certain chronic conditions) and Medicaid (primarily targeted at low-income patients).

The patient had not been employed for several years before presenting at our institution. As a result, he was unable to access private health insurance through an employer. However, the patient qualified for federal Medicare programs, through which he was only partially enrolled. The patient was enrolled in and covered by Medicare Part A, the portion of federally funded health insurance covering inpatient hospital care, skilled nursing facilities, hospice, lab tests, surgery, and home health care. On the other hand, the patient was not covered by Medicare Part B, the component of federal health insurance related to doctor’s services, outpatient care, and medical services that do not fall under the purview of Medicare Part A. Although the Medicare Savings Program covers premiums, deductibles, coinsurance, and copayments through Part B, only about half of the eligible seniors are enrolled [15].

This lack of thorough health insurance, and the high out-of-pocket cost of any procedure or treatment, discouraged the patient from seeking medical care for his wound. If the lesion had been addressed early in the disease process, the likely treatment would have consisted of an in-office procedure with simple and successful tumor removal. However, due to the patient’s inability to pay any out-of-pocket costs for these early treatments, an extensive and exophytic lesion of the scalp developed and continued to grow. As opposed to a simple one-time procedure and office follow-up, this more advanced tumor resulted in five ED visits over the span of two months, repeat wound care clinic appointments, a major excisional surgery, and ongoing follow-up visits and cancer screenings.

One important component of developing a treatment plan for this patient was the development of an optimal pre-operative plan by taking the time pre-operatively to assess the extent of the tumor accurately. The determination was made to treat the patient’s lesion with a wide local excision. In addition to the primary surgical procedure, the care team also had to decide upon the most appropriate strategy for wound closure following the excision; this decision was made through consideration of the scalp reconstructive ladder. The reconstructive ladder approach generates a hierarchy of increasingly complicated graft procedures to select the least complicated procedure on the ladder that will sufficiently treat the patient [16]. The lowest level of this procedural hierarchy is healing via secondary intention, in which the physician maintains a clean wound site and allows the healing process to occur on its own [17]. This approach has the benefit of minimizing intervention; however, it may result in longer healing times or alopecia [18].

Additional reconstructive options can include primary closure, skin grafting, local and regional flaps, and free tissue transfer. Primary closure is a relatively straightforward and time-efficient procedure; however, it is usually limited to smaller defect sizes. Local flaps offer good coverage and healing of scalp defects, but in our patient’s case, the anticipated defect was deemed too large for local flap advancement or rotation. Regional flaps have the disadvantage of only reaching the occipital or temporoparietal areas, and in our patient’s case, his surgical defect was midline at the vertex. The highest level of the scalp reconstructive ladder is a free flap or free tissue graft, in which a large vascularized tissue is transferred to the surgical defect site, and microvascular anastomosis is performed [18,19]. Generally, it is recommended to choose the least complex method to achieve optimal reconstruction or coverage of the surgical defect. In our case, we opted for a split-thickness skin graft. Harvesting the split-thickness skin graft is a quick and efficient procedure, and when placed on an adequately prepared defect site, the graft offers reliable healing. One final consideration in our patient was the area of the exposed calvarium. Every effort needs to be made to cover exposed bone. In this case, the patient had a 5 x 5 cm area of the exposed bone secondary to resection of pericranium due to involvement by disease. Carefully burring the outer table of the exposed bone until encountering bleeding from exposed capillaries is generally sufficient to adequately prepare the surgical defect site to receive a split-thickness graft.

This case highlights the importance of pre-operative evaluation and planning, along with the consultation of a multidisciplinary tumor board. Having a multidisciplinary team provides the benefit of interprofessional decision-making that improves multiple elements of cancer care, including patient survival outcomes [20]. In this patient's case, the discussion initially centered on treatment options and planning. The group was consulted on the management of the neck in this patient with a midline SCC lesion. The two potential treatment approaches were to perform bilateral parotidectomies and modified radical neck dissections or to undergo close surveillance for lymph node involvement in the postoperative period with a neck ultrasound every three months. By taking the time pre-operatively to assess the extent of the tumor accurately, we were able to prevent overtreatment and an increased surgical burden for this patient.

Since the patient did not present with evidence of lymph node involvement on either physical examination or imaging, the decision was made to proceed with close surveillance of the neck following surgery. In addition, the patient was once again presented at the tumor board to discuss the indication of adjuvant treatment. Since pathology demonstrated clear margins with no adverse tumor features, there was no strong indication to proceed with adjuvant radiation. The decision was made to continue with close surveillance of the primary site and neck with clinical examination and neck ultrasounds. Ultimately, the selection of the best treatment path for this patient was aided by consultation with the institutional tumor board, which provided expert multidisciplinary advice.

## Conclusions

This article presents the case of a 68-year-old male with an extensive and exophytic SCC of the scalp. In addition to predisposing factors, such as diabetes and smoking, this tumor was neglected due to financial constraints, which allowed the lesion to develop and eventually require extensive surgical intervention. Despite the delay between the formation of the nonhealing ulcer and the initial presentation of the patient for medical treatment, it was still possible to perform surgical resection with curative intent. This surgical resection was successfully performed with no need for adjuvant therapies, and the patient is currently on follow-up with no recurrence of the disease.
